# Modification of α-synuclein by lipid peroxidation products derived from polyunsaturated fatty acids promotes toxic oligomerization: its relevance to Parkinson disease

**DOI:** 10.3164/jcbn.18-25

**Published:** 2018-05-01

**Authors:** Masayo Shamoto-Nagai, Shinsuke Hisaka, Makoto Naoi, Wakako Maruyama

**Affiliations:** 1 Department of Health and Nutrition, Faculty of Psychological and Physical Science, Aichi Gakuin University, 12 Araike, Iwasaki-cho, Nissin, Aichi 470-0195, Japan; 2 Graduate School of Pharmacy, Meijo University, 150 Yagotoyama, Tenpaku-ku, Nagoya, Aichi 466-8503, Japan

**Keywords:** α-synuclein, lipid hydroperoxide, mitochondria, oligomerization, Parkinson disease

## Abstract

Recently, toxic α-synuclein oligomer, which can mediate cell-to-cell propagation is suggested to cause sporadic Parkinson disease. α-Synuclein interacts with membrane lipids especially polyunsaturated fatty acids to stabilize its three-dementional structure. Peroxidation of polyunsaturated fatty acids may reduce their affinity to α-synuclein and peroxidation byproducts might modify α-synuclein. 4-Hydroxy-2-nonenal derived from *n*-6 polyunsaturated fatty acids was reported to modify α-synuclein to produce a toxic oligomer. Moreover, the accumulation of 4-hydroxy-2-nonenal, which could induce oligomeriztion of α-synuclein, was found in parkinsonian brains. Docosahexaenoic acid, an *n*-3 polyunsaturated fatty acids abundant in the neuronal membrane, was also found to enhance α-synuclein oligomerization; however, the precise details of the chemical reaction involved are unclear. Propanoylated lysine, a specific indicator of docosahexaenoic acid oxidation, was increased in neuronal differentiated human neuroblastoma SH-SY5Y cells overexpressing α-synuclein. α-Synuclein might be modified by the peroxidation products and then, is degraded by the autophagy-lysosome system. In addition, in the cells overexpressing α-synuclein, the mitochondrial electrone transfer chain was found to be inhibited. Accumulation of abnormal α-synuclein modified by lipid radicals derived from polyunsaturated fatty acids may be not only an indicator of brain oxidative stress but also causative of neurodegeneration such as Parkinson disease by impairing mitochondrial function.

## Introduction

Histopathological characteristics in neurodegenerative disorders, including Alzheimer disease (AD), Parkinson disease (PD) and Huntington disease, are loss of a distinct population of neurons and the accumulation of disease-specific aberrant proteins. Oxidative stress, mitochondrial dysfunction, and impaired cellular systems to cleave modified, denatured proteins, such as the ubiqutin-proteasome system (UPS) and the autophagy-lysosome system, have been proposed as the major pathogenic factors in these disorders.^(1–5)^

**Fig. 1:**
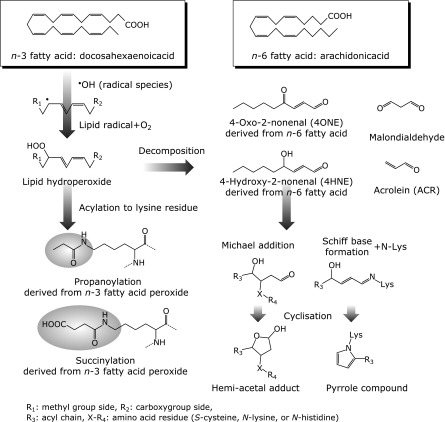
Oxidation pathways of *n*-3 and *n*-6 PUFAs. PUFAs can be easily oxidized by radical species such as hydroxyl radical and superoxide anion radical, then, producing lipid hydroperoxides. Hydroperoxides can form amide type adducts on the ε-amino group in lysine residue. Peroxides from *n*-3 family such as docosahexaenoic acid (DHA) induce propanoylation as an acylation with three carbons. Low molecule aldehydes such as malondialdehyde and acrolein are generated through further decomposition of lipid hydroperoxide. 4-hydroxy-2-nonenal (4HNE) and 4-oxo-2-nonenal (4ONE) are well-known electrophilic aldehydes produced by *n*-6 PUFAs oxidation. 4HNE reacts to some amino acids, cysteine, the imidazole group of histidine and the ε-amino group of lysine (as Michael addition on carbon 3 in 4HNE). Adduct formation of 4HNE gives a hemiacetal structure. In addition, 4HNE can bind covalently to primary amino group in amino acid, which forms a Schiff base showing the reaction between 4HNE and lysine residue to produce pyrrole compound via cyclization.

PD is the second most common neurodegenerative disorder in the elderly after AD, with a prevalence of about 0.3% of the population in industrialized countries. The incidence increases with age to 4% of the population over 80 from 1% in that over 60-years of age, suggesting that aging is the major risk factor. The main symptoms of PD are rigidity, tremor, bradykinesia, and instability, which are ascribed to the degeneration of nigral dopaminergic neurons.^(6,7)^ Dopamine is oxidized enzymatically by monoamine oxidase with the production of hydrogen peroxide (H_2_O_2_), or non-enzymatically into dopamine quinone and superoxide (O_2_^−^). These radicals and hydroxyl radical (OH^•^) are potent cytotoxic reactive oxygen species (ROS). Nitric oxide (NO) is produced from l-arginine by inducible nitric oxide synthase in microglia and astrocytes and taken up into neurons. NO reacts with O_2_^−^ produced by dopamine oxidation to generate highly reactive peroxynitrite (ONOO^−^), one of the reactive nitrogen species (RNS). ONOO^−^ is a strong oxidant for modifying tyrosine residues in protein to 3-nitrotyrosine. Therefore, dopamine neurons are inevitably exposed to oxidative stress, which may be responsible for the age-related decline in the number of neurons.^(8,9)^ The accumulation of oxidative injury in dopamine neurons was confirmed in PD brain as shown by the increase in oxidatively modified proteins such as nitrotyrosine,^(10)^ 4-hydroxy-2-nonenal (4HNE),^(11)^ and acrolein (ACR).^(12)^ A pathological hallmark in PD brain is the protein inclusions in the degenerating dopamine neurons, called Lewy bodies, whose main component is α-synuclein (αSyn). 4HNE and ACR are lipid aldehydes derived from polyunsaturated fatty acids (PUFAs) in the phospholipid components of the biomembrane (Fig. 1). The proteins modified by these aldehydes were detected in the mitochondrial electron transport chain (ETC), especially complex I, suggesting the association of oxidative stress with complex I deficiency, a pathogenic factor for PD.^(13,14)^

The point mutations of αSyn, A53T, A30P and E46K^(15–17)^ and the duplication or triplication of the normal gene^(18)^ has been proved to cause the familial forms of PD. The observation that patients with a triplication have earlier onset and faster progression than the patients with a duplication suggests a that gene dosage effect of the αSyn gene.^(19)^ In addition, genome-wide association studies^(20,21)^ also indicated that αSyn is involved in the sporadic form of PD. It is highly possible that a slight but persistent increase in the level of αSyn induces neuronal death in the sporadic form of PD. In addition, oxidative modification of αSyn may induce three-dementional conformational change of the protein and then, may increase its toxicity. Post-translational modification of αSyn regulates its tertiary structure and toxicity, in addition to its concentration and genetic modulation, that is, gene mutation.^(22)^ This paper reviews the possible involvement of the modification of αSyn by peroxidation products of PUFAs in the pathogenesis of PD.

## Characteristics of Toxic αSyn Relating Parkinson Disease

The sequence of αSyn (14-kDa) is composed of an amphipathic lysine-rich amino terminus interacting lipid membrane, a hydrophobic central region called NAC (non-amyloid beta component), which initiates protein aggregation, and a highly acidic C-terminal. αSyn aggregation from monomers via intermediate oligomers into amyloid fibrils is considered to be the causative process of the PD pathology. The toxicity of artificial αSyn mutant that promotes oligomers or fibrils was studied using a rat lentivirus system. It was shown that αSyn oligomer was toxic to rat dopamine neurons but the fibrillar form was not *in vivo*.^(23)^ The mechanism of the toxicity was investigated, and it was found that membrane-associated αSyn oligomer disrupted the membrane to increase the permeability. In contrast, aggregation of αSyn may be neuroprotective by sequestering its toxic oligomer. The Lewy body load in PD patients was found to be poorly correlated with the severity of the symptoms,^(24)^ and widespread and abundant Lewy body pathology was detected even in older people without neurodegenerative disease.^(25)^ The human pathology findings indicate that αSyn inclusions may represent detoxified pathway in α-synucleinopathy.

## Modification of αSyn by Lipid Radicals Derived from PUFAs May Promote Toxic αSyn Oligomerization

The membrane lipid peroxidation hypothesis in aging is widely accepted but its biological background has not been fully elucidated.^(26)^ αSyn is abundant in the brain and the presence of its lipid-binding motif at the *N*-terminal of the protein suggests its interaction with phospholipid composition in the membrane.^(27)^ αSyn shows high affinity to PUFA and its interaction lipids stabilizes its α-helical structure.^(28)^ ROS damage the neuronal membrane and can initiate lipid peroxidation of PUFAs such as docosahexaenoic acid (DHA) and arachidonic acid (ARA). Such reactions initiate the formation of reactive aldehydes, such as 4-oxo-2-nonenal (4ONE), 4HNE, ACR and malondialdehyde (Fig. 1). 4ONE and 4HNE, the major lipid peroxidation byproducts of PUFAs were found to modify αSyn covalently and produce oligomers *in vitro*.^(29)^ Moreover, 4ONE- and 4HNE-induced oligomeric αSyn was found to be toxic to human neuroblastoma SH-SY5Y cells, but native αSyn was not.^(30)^ In another study, differentiated SH-SY5Y cells overexpressing αSyn were treated with 4HNE. 4HNE-induced αSyn oligomer (4HNE-oligomer) was found to mediate cell-to-cell transfer not only by increasing secretion through exosomes, but also increased intracellular internalization of the oligomer.^(31)^ Prion-like propagation of αSyn is now drawing attention as a key characteristic of toxic αSyn oligomer.^(32)^ The presence of 4HNE-oligomer was investigated using human postmortem brain. The specific monoclonal antibody against 4HNE-oligomer was found to have strong affinity to Lewy body-like structures in the brains from PD and diffuse Lewy body disease, but no-staining was detected in no-neurological control subjects. Furthermore, in the brain of (Thy-1)-h[A30P]-αSyn transgenic mice, the accumulation of 4HNE-oligomer was detected with aging.^(33)^ However, the mechanism behind the neurotoxicity of 4HNE-oligomer was still enigmatic. Recently, 4HNE-oligomers were reported to be taken up by astrocytes to damage mitochondrial function, and the antibodies to αSyn oligomers ameliorated the dysfunction.^(34,35)^

The synthesis of 4HNE has been mainly ascribed to the oxidation of *n*-6 PUFA (Fig. 1). ON the other hand, DHA (*n*-3 PUFA) is the most abundant PUFA in the human brain and believed to be neuroprotective, but its oxidation may produce harmful lipid radicals/products. It is suggested that αSyn forms a covalent adduct with the oxidation product of PUFAs, especially DHA, along with the scavenging of harmful lipid radicals.^(36)^ On the other hand, DHA may enhance the formation of αSyn stable oligomer and exhibit the toxicity by increasing membrane permeability.^(37)^ Indeed, the exposure of mesencephalic neurons of αSyn transgenic mice to PUFAs (ARA and DHA) was shown to enhance cytosolic αSyn oligomerization.^(38)^

Hydroperoxides are early products of lipid peroxidation before aldehyde production, and the protein modification by hydroperoxide can be detected by using specific antibodies.^(39)^ It was found that alkylamide and carboxylalkylamide derivatives of DHA make stable adducts with lysine residues in the proteins as *N*^ε^-propanoyl-lysine (PRL) and *N*^ε^-succinyl-lysine (SUL), respectively.^(40)^ Then, PRL and SUL are considered to be specific markers of the DHA-derived modification of proteins. To detect SUL, hydrolysis of the ester bond is required because most of the carbonyl terminal of SUL is occupied by cholesterols or phospholipids. The detection of PRL is the easier indicator of DHA oxidation as opposed to SUL, but no reports have been published on the detection of PRL-immunoreactive proteins in neurodegenerateive disorders.

## αSyn Overexpression Increased Proteins Modified by DHA-Derived Hydroperoxide and Impaired Mitochondrial Function

Mitochondrial dysfunction is closely related to the pathogenesis of PD. Complex I protein in ECT was found to be decreased in PD brain.^(41,42)^ A recent paper reported that a considerable amount of αSyn is located in the mitochondrial associated membrane (MAM), the mitochondrial membrane domain contacting with endoplasmic reticulum (ER) membrane.^(43)^ MAM is now drawing attention as a regulator of calcium homeostasis by transferring calcium between ER and mitochondria. αSyn overdose was suggested to disrupt MAM function. Perturbation of intracellular calcium homeostasis is suspected to cause mitochondrial dysfunction, activation of apoptotic signal, ER stress, and increased membrane lipid peroxidation.

**Fig. 2:**
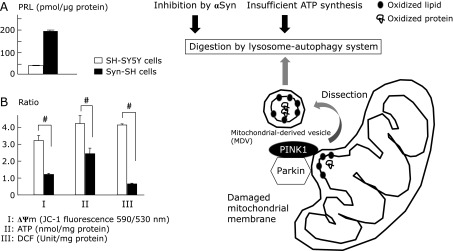
ROS leaked from mitochondrial ETC damage membrane lipid and proteins to decrease mitochondrial membrane potential (ΔΨm). Parkin and PINK1, which cause autosomal recessive PD, detect the membrane with low ΔΨm and dissect the membrane. Mitochondrial derived vesicle (MCV) is digested by autophagy-lysosome system. The mitochondrial function was estimated using neuronal differentiated wild SH-SY5Y cells and SH-SY5Y cells transfected with αSyn (Syn-SH cells) (A) Overexpression of αSyn decreased ΔΨm, ATP content, and ROS/RNS production as shown by 2',7'-dichlorodihydrofluorescein (DCF) production. Open column: control SH-SY5Y cells, filled column: Syn-SH cells. The column and the bar represent mean and SD of 3 independent experiments. ^#^*p*<0.01. (B) In Syn-SH cells, accumulation of proteins positive for PRL was detected by ELISA method. Open column: control SH-SY5Y cells, filled column: Syn-SH cells. αSyn may directly inhibit autophagy-lysosome system or indirectly through mitochondrial dysfunction and as a result, PRL-positive proteins accumulated in the cells.

Another mechanism by which αSyn impaires mitochondrial dysfunction is through inhibiting the removal of damaged mitochondria. Disregulation of mitochondrial quality control is proposed to be a pathological process in PD. Mitochondria are the organelles that generate energy in cells but simultaneously, with this undergo ROS leakage from the ETC, which can damage the surrounding biomolecules, especially mitochondrial membrane lipids. Damaged mitochondrial membrane with reduced mitochondrial membrane potential (ΔΨm) can be detected by assaying PINK1 and Parkin, both of which are known to be responsible for autosomal recessive familial PD. Massive damage to the mitochondria was shown to be removed by mitophagy and localized damaged membrane was dissected to form mitochondrial-derived vesicles (MDVs).^(43,44)^ These “bad” mitochondria are digested by macroautophagy or the autophagy-lysosome system (Fig. 2). The native αSyn was suggested to be degraded by chaperone-mediated autophagy and macroautophagy in neuronal cells, and macroautophagy activity was found to be inhibited by αSyn.^(45,46)^ The mitochondrial dysfunction induced by αSyn should be investigated in more detail in relation to mitochondrial quality control and inhibition of the autophagy-lysosomal system.

We studied whether the accumulation of peroxidation products derived from DHA is truly increased by αSyn. Our recent experiment demonstrated that after co-incubation of DHA with αSyn, oligomerization of αSyn, which is immunoreactive to a PRL antibody, was detected *in vitro* (Shamoto-Nagai *et al.*, in preparation). As shown in Fig. 2A, the increase of PRL-containing proteins was clearly shown in human neuroblastoma SH-SY5Y cells overexpressing αSyn (Syn-SH cells). Some of the PRL-modified proteins in SH-SY5Y cells overexpressing αSyn may be αSyn adducted with DHA-derived peroxides but the issue requires further investigation. It is not certain whether αSyn scavenges hydroperoxides derived from DHA and protects the cells from oxidative stress, or produces toxic and modified αSyn oligomers. In our system, decreased ATP synthesis, ΔΨm, and ROS-RNS level were observed in αSyn-overexpressing cells, indicating the inhibition of mitochondrial ECT (Fig. 2B). The amount of PRL-modified proteins was increased in the cells although the level of ROS-RNS was significantly decreased. The most likely explanation for this findings is that proteolysis machinery for oxidatively modified proteins (including PRL-modified proteins) was reduced because of insufficient ATP synthesis.

**Fig. 3:**
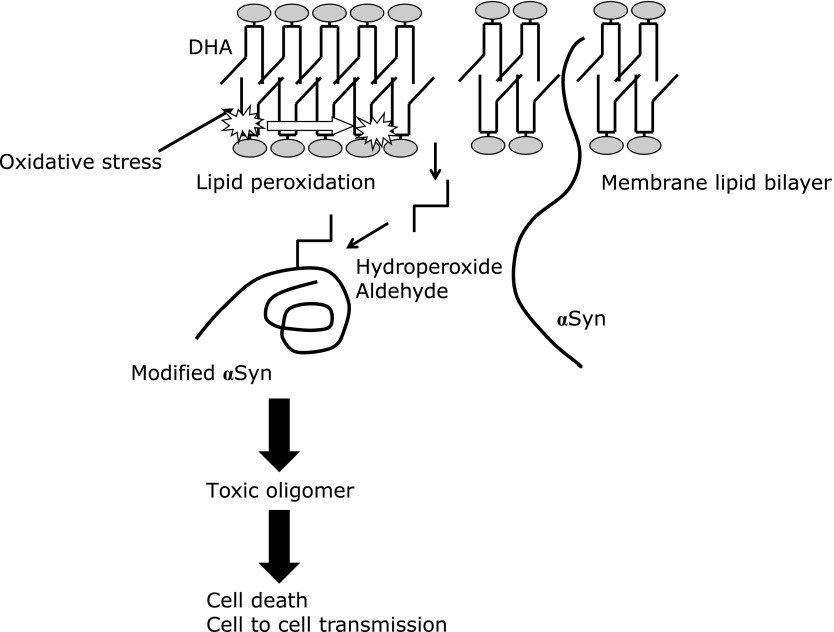
αSyn makes adduct with lipid peroxide and aldehyde derived from membranous DHA to produce toxic oligomer. αSyn oligomer induces neurodegeneration by perturbation of mitochondrial function. αSyn modified by lipid aldehyde may seed on prion-like propagation.

The mechanism of cell death in PD has not been clarified. Mitochondrial defects, ER stress, inhibition of UPS, glial inflammation, membrane damage, autophagy-lysosomal dysfunction, and synaptic destruction have all been suggested to be involved. These cellular dysfunctions are not independent, but should instead form vicious cycle to induce cell death. Lipid peroxides derived from PUFAs may be not only indicators of brain aging but also causative factors of PD by promoting aberrant αSyn in the brain (Fig. 3).

## Abbreviations

ACR: acrolein
AD: Alzheimer disease
αSyn: α-synuclein
ARA: arachidonic acid
ΔΨm: mitochondrial membrane potential
DHA: docosahexaenoic acid
ETC: electrone transfer chain
4HNE: 4-hydroxy-2-nonenal
MAM: mitochondrial associated membrane
4ONE: 4-oxo-2-nonenal
PD: Parkinson disease
PRL: propanoyl-lysine
RNS: reactive nitrogen species
ROS: reactive oxygen species
SUL: succinyl-lysine
UPS: ubiqutin-proteasome system
